# Examination of GFAP Among a Racial and Ethnically Diverse Community‐Based Cohort: An HABS‐HD Study

**DOI:** 10.1002/alz.089498

**Published:** 2025-01-09

**Authors:** Melissa Petersen, Fan Zhang, James Hall, Sid E. O'Bryant, the HABS‐HD Study Team

**Affiliations:** ^1^ University of North Texas Health Science Center, Fort Worth, TX USA

## Abstract

**Background:**

Rates of Alzheimer’s disease (AD) Dementia are expected to grow considerably among certain ethnic/racial groups, including Blacks and Hispanics. Glial fibrillary acidic protein (GFAP) has been examined as a biomarker of AD; however, limited research has been conducted examining this particular marker among diverse populations. Therefore, this study sought to address this gap and provide an initial examination among a diverse community‐based cohort.

**Method:**

Participants included n= 326 (n=125 Black; n=26 Hispanic; n=175 non‐Hispanic white) from the Health and Aging Brain Study‐Health Disparities. Clinical information, including age, education, and medical history, was derived via a clinical interview. Cognitive diagnosis of Mild Cognitive Impairment and Dementia were determined by consensus review. Plasma samples were assayed for GFAP using Quanterix kits and run on an HD‐X imager. PET Tau neuroimaging included the ^18^F‐PI‐2620 tracer. PET Tau positivity was determined based on a clinical read. Statistical analyses were run across racial/ethnic groups and included a correlational analysis between GFAP and PET Tau positivity. Additional analyses included examining the relationship between GFAP, demographic factors, medical comorbidities, and cognitive diagnosis. Statistical significance was set at p<0.05.

**Result:**

A significant correlation was found between GFAP and a clinical read of PET Tau positivity only among Blacks (p‐value =0.002). Black participants were also found to have a significant relationship between GFAP and age (p‐value<0.001) as well as a cognitive diagnosis of Dementia (p‐value=0.002). Among non‐Hispanic whites, GFAP was found to be significantly related to age (p‐value=0.03) and a medical diagnosis of Diabetes (p‐value=0.003). No significant findings were found between GFAP and variables such as education, hypertension, dyslipidemia, or a cognitive diagnosis of Mild Cognitive Impairment. There were no significant findings identified among Hispanics.

**Conclusion:**

The link between GFAP and select demographic and cognitive factors was found to be greater among Blacks and non‐Hispanic whites; however, findings were likely limited among Hispanics due to the relatively small sample size. Although there was a limited number of participants who were PET Tau positive, the only significant correlation found was among Blacks, highlighting the further need examine such AD biomarkers among diverse populations.

